# Comparison of Functional Outcomes of Superficial Palmar Branch of Radial Artery (SPBRA) and Free Venous Flap (FVF) Techniques for Finger Tissue Defects

**DOI:** 10.3390/jcm14020310

**Published:** 2025-01-07

**Authors:** Numan Atilgan

**Affiliations:** Department of Hand Surgery, Private Clinic, Gaziantep 27060, Turkey; doktor_dao@hotmail.com; Tel.: +90-507-221-19-45

**Keywords:** finger tissue defects, free flap surgery, SPBRA free flap, free venous flap, flap survival rates

## Abstract

**Objectives**: This study aimed to compare two surgical techniques—the free flap of the superficial palmar branch of the radial artery (SPBRA) and the free venous flap (FVF)—to evaluate their efficacy and aesthetic outcomes in repairing finger tissue defects. The goal was to determine which procedure offers faster healing curves and better overall patient outcomes, ultimately improving the quality of life for individuals undergoing these surgeries. **Materials and Methods**: A retrospective study was conducted using the clinical database of Sanliurfa Mehmet Akif Inan Education and Research Hospital, University of Health Sciences, from 1 January 2019 to 1 January 2022. A total of 44 patients with finger tissue defects, excluding thumb defects, were randomly divided into two groups: 21 patients underwent the SPBRA free flap procedure and 23 patients underwent the FVF procedure. Primary endpoints included flap survival rates, sensory recovery rates, aesthetic satisfaction scores, and complication rates. Data were collected during preoperative assessments and postoperative follow-ups at 1, 3, and 6 months. **Results**: The SPBRA group demonstrated a higher success rate (95% vs. 92%) and greater patient satisfaction in terms of restoring normal appearance and function. The SPBRA technique also showed superior sensory recovery with a lower two-point discrimination score (mean SPBRA = 6 mm vs. mean FVF = 8 mm). Functional outcomes, assessed by the Michigan Hand Outcomes Questionnaire, indicated higher scores for the SPBRA group (85/100) compared to the FVF group (80/100). Additionally, the SPBRA procedure was associated with fewer complications, highlighting its effectiveness and safety. **Conclusions**: The findings suggest that the SPBRA free flap technique offers better outcomes than the FVF procedure for repairing finger tissue defects. It provides superior functional restoration, enhanced cosmetic satisfaction, and a lower rate of complications. These results support the preference for the SPBRA technique in addressing complex finger tissue defects and improving patient outcomes.

## 1. Introduction

Finger tissue defects caused by occupational accidents represent a significant healthcare challenge, resulting in substantial functional impairment, loss of productivity, and decreased quality of life [1,2]. These injuries often necessitate advanced surgical interventions to restore functionality and aesthetics. Among the various methods developed, free flap techniques have emerged as a significant advancement in reconstructive surgery, offering high success rates and improved recovery outcomes [3,4].

The superficial palmar branch of the radial artery (SPBRA) flap and the free venous flap (FVF) are two widely used techniques for repairing finger tissue defects. SPBRA provides enhanced vascularization and aesthetic results due to its robust blood supply, while FVF is considered less invasive and more suitable for smaller defects [5,6]. The SPBRA technique has been increasingly favored in clinical practice because it combines reliable vascularization with the potential for superior sensory recovery. This approach has demonstrated significant improvements in both functional and aesthetic outcomes, leading to faster healing and reduced complication rates compared to other flap techniques [7].

On the other hand, the FVF method is known for its simplicity and the ability to cover a wide range of defect sizes. Despite its advantages, FVF has shown variability in sensory recovery and higher rates of complications in larger defects [8,9]. The literature comparing the two techniques highlights the need for clearer evidence regarding their efficacy in different clinical scenarios. While some studies suggest SPBRA’s superiority in terms of flap survival and sensory restoration, others report similar outcomes between the two techniques, underscoring the importance of patient-specific factors and surgical expertise [10,11,12].

This study aims to evaluate and compare the functional and aesthetic results of SPBRA free flap versus FVF techniques in finger tissue defects. The specific objective is to identify the technique that leads to better vascularization, rapid healing, and improved sensory recovery, so that it can become the preferred clinical method.

## 2. Materials and Methods

### 2.1. Study Design and Study Population

This study was designed as a retrospective comparative study. It covers the period from 1 January 2019 to 1 January 2022 and was carried out in the Department of Orthopedics and Traumatology at the Mehmet Akif nan Training and Research Hospital. Ethical approval from the committee was obtained, and all patients provided written informed consent. This research involved 44 patients with finger tissue defects. Participants were stratified into two major groups: those treated with a free flap of the SPBRA and those with FVF.

Each patient’s finger tissue defect was recorded based on its size and location, categorized as being at the fingertip, middle, or lower parts of the finger. Measurements in millimeters classified the defects as minimal (1–10 mm), moderate (11–20 mm), or large (>20 mm). For both groups, flaps were harvested from wrist locations. In the SPBRA group, one artery and two veins were anastomosed, while in the FVF group, anastomosis involved one afferent vein and one efferent vein. The two techniques were then compared in terms of post-treatment complications, as well as functional and cosmetic outcomes.

The inclusion criteria for this study were male and female patients aged 18 years and older, with tissue defects in the fingers, excluding those involving the thumb, and suitable anatomical and health conditions for SPBRA or FVF surgery. Exclusion criteria included patients with active infections, serious systemic diseases such as uncontrolled diabetes, cancer, or chronic kidney disease, impaired wound healing from previous surgical interventions at the same site, coagulation disorders, or those on anticoagulant therapy. Patients who refused to participate, did not provide written informed consent, or were active smokers due to the known negative impact on wound healing were also excluded.

Of the 44 patients included in the study, 21 underwent the free flap procedure using SPBRA, while 23 received FVFs. This study evaluates the functional and aesthetic outcomes achieved with SPBRA compared to FVFs. Patients were randomly assigned to either group upon their initial hospital visit, and preoperative and postoperative evaluations were conducted using standard methodological approaches. All procedures adhered to the principles of the Declaration of Helsinki and were approved by the institutional ethics committee. Patients were followed for a minimum of six months. Data were collected preoperatively, perioperatively, and during scheduled follow-up visits at one, three, and six months postoperatively. Operative records were also reviewed up to six months after discharge.

### 2.2. Surgical Technique

In this study, two surgical procedures were used in patients with finger tissue defects: free flap from the SPBRA and FVF. The FVF was harvested from the dorsal venous system of the hand, ensuring adequate vein length and diameter for anastomosis. The use of an axillary brachial plexus block significantly eased pain at the site of surgery and improved patient comfort. The SPBRA technique involves harvesting a free flap from the superficial palmar branch of the radial artery, which provides robust vascularization to the transferred tissue. The procedure begins with the identification and dissection of the superficial palmar branch at the wrist level. The flap is carefully designed based on the defect size and shape, ensuring adequate perfusion. Microsurgical techniques are used to perform anastomoses, typically involving one artery and two veins, to establish optimal blood flow. The harvested flap is then transferred to the finger tissue defect site and secured with sutures. This technique is particularly suited for larger defects requiring enhanced vascular supply and allows for excellent functional and aesthetic outcomes due to its ability to promote tissue viability and sensory recovery. Anastomosis between an efferent and afferent vein was performed in an FVF procedure. Both techniques involved the placement of flaps using microsurgical techniques to ensure perfusion to tissues.

All surgical procedures in both groups took place under aseptic conditions, either under general or local anesthesia. After surgery, analgesics and antibiotics were administered for pain control during recovery and infection prevention, respectively. Close follow-up was maintained after operation regarding flap viability and functional recovery processes. The location and size determined the treatment results. The second finger was treated more frequently than any other fingers, while the fifth finger was treated less frequently than all others. Proper rehabilitation processes were considered important for better functional returns.

### 2.3. Measurements

In this study, we collected various types of data to comprehensively evaluate the outcomes of SPBRA and FVF techniques. Demographic information, including age, gender, and occupation type, was recorded for all participants. The functional outcomes assessed included flap survival rates, sensory recovery (measured by static and mobile two-point discrimination (s2PD and m2PD) tests, as well as Semmes–Weinstein monofilament (SWM) testing), and aesthetic satisfaction, which was rated using a visual analog scale. Additionally, the Michigan Hand Outcomes Questionnaire was used to evaluate functional recovery and daily activity performance. Complications such as infections, hematomas, flap loss, and joint contractures were also documented to assess the safety of each procedure. These data points provided a comprehensive understanding of the efficacy and impact of the surgical techniques under investigation.

Subjective assessments include cold intolerance, aesthetic appearance, and functional recovery. According to patients, a repaired finger was rated according to daily activities and severity of pain (no, mild, moderate, severe). Furthermore, subjective cosmetic appearance and pain at the donor site and reconstructed finger were assessed by patients with a visual analog scale, which ranges from 0–10 (lowest satisfaction—highest satisfaction).

### 2.4. Statistical Analysis

To compare the results of the two surgical methods, statistical tests were performed. For example, Student’s T-test was used to analyze continuous variables represented by numerical data, such as functional recovery scores and aesthetic satisfaction ratings. Sensory improvement measures such as the results of the s2PD and m2PD tests were also subjected to this test. On the other hand, ordinal or ranked data that include cold intolerance ratings and SWM test results were evaluated using the Mann–Whitney U test. These statistical significances between two groups indicate that there are significant differences if *p* < 0.05.

## 3. Results

In this study, 44 patients were analyzed, with 21 undergoing the SPBRA procedure and 23 receiving FVFs. The mean age was 34 years in the FVF group and 36 years in the SPBRA group, with an age range of 18 to 57 years for both groups. Regarding gender distribution, all patients were male except for one female in the SPBRA group (twenty males and one female) and one female in the FVF group (twenty-two males and one female). There were no statistically significant differences between the two groups in terms of age, sex, or the number of treated fingers (*p* > 0.05). Most patients in both groups had only one finger treated, while cases involving two or three fingers were less common (Table 1).

Operations in the SPBRA group lasted an average of 140 to 190 min, depending on the defect size. In the FVF group, the duration ranged from 130 to 175 min, also based on the size of the defect. Both groups utilized general and local anesthesia during the procedures. In the SPBRA group, fifteen patients underwent surgery under general anesthesia, while six were operated on under local anesthesia. In the FVF group, eighteen patients received general anesthesia, and five underwent surgery under local anesthesia. The flap survival rates varied according to defect size. For the SPBRA group, the survival rates ranged from 100% to 80%, depending on the defect dimensions, while similar rates were observed in the FVF group (100% to 80%). One patient in the SPBRA group experienced flap loss, primarily due to poor circulation leading to ischemia. This group also reported two cases of infection and one hematoma. In the FVF group, there was one case of flap loss, three cases of infection, two hematomas, two joint contractures, and two instances of keratolysis (Table 2).

The Michigan Hand Outcomes Questionnaire was used to evaluate functional recovery in both groups. The SPBRA group recorded a mean score of 85 out of 100, while the FVF group achieved an average score of 80 out of 100. Mobility, grip strength, and sensory recovery showed better outcomes in the SPBRA group compared to the FVF group. This was reflected in a higher level of aesthetic satisfaction among SPBRA patients, whereas FVF patients reported more complaints related to cold intolerance (Table 3).

Static and mobile two-point discrimination tests, used to measure sensory recovery, demonstrated that the SPBRA group achieved better results compared to the FVF group. In the static two-point discrimination test, the SPBRA group had a mean value of 6 mm, while the FVF group recorded 8 mm. For the moving test, the SPBRA group measured 4 mm, compared to 6 mm in the FVF group. Additionally, the Semmes–Weinstein monofilament test revealed a sensory threshold reduction of 2.83 g in the SPBRA group, indicating improved sensory recovery (Table 4).

There were significant statistical differences between the SPBRA and FVF groups. The SPBRA group scored higher in terms of functional restoration and aesthetic satisfaction compared to the FVF group. However, this distinction did not apply to cold intolerance, where the FVF group reported higher values than the SPBRA group. Additionally, the comparison between the two groups showed statistical significance (*p* < 0.05), indicating that the SPBRA group achieved better sensory recovery rates on average (Table 5).

**Figure 1:**
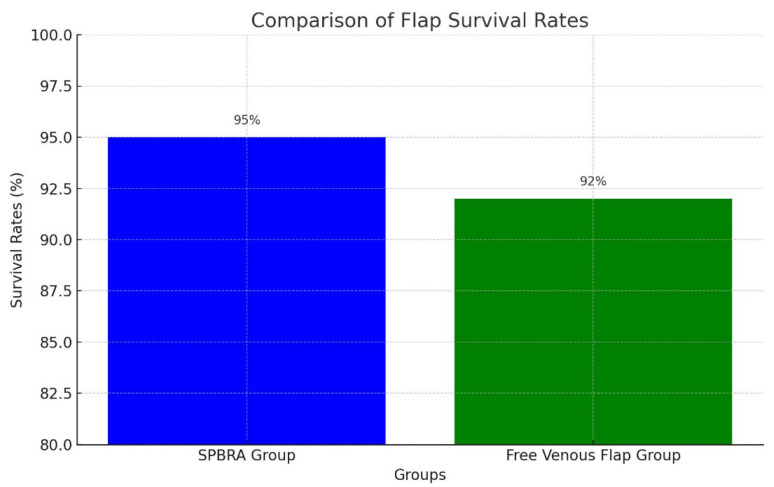
Comparison of flap survival rates.

The results of the surgical interventions revealed a clear contrast between the two groups. Procedures performed in the SPBRA group outperformed those in the FVF group, with the SPBRA group achieving approximately three points higher in functional outcomes and a 95% success rate in terms of flap survival (Figure 1).

**Figure 2:**
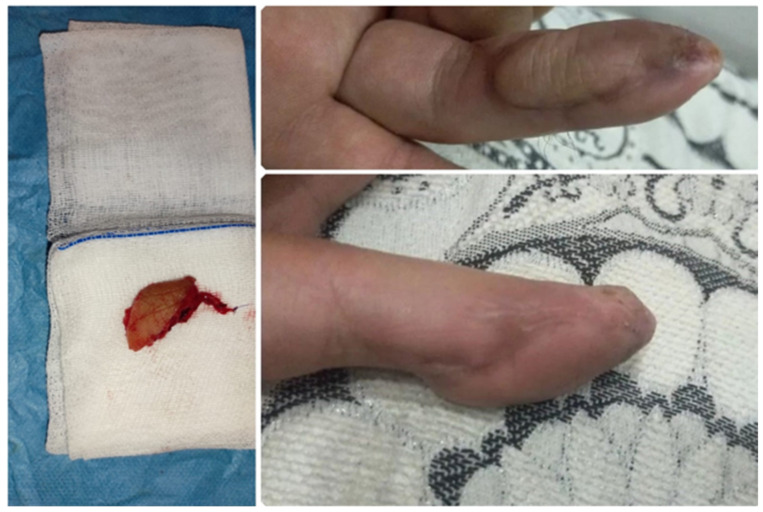
Surgical procedure and flap excision and postoperative recovery (six months after surgery).

Additionally, in terms of pain and aesthetic satisfaction, the SPBRA group reported lower pain levels and higher satisfaction scores, indicating greater comfort and satisfaction among patients after treatment. Healing processes, recorded from the first months of therapy, showed improvement over time in both groups; however, the SPBRA group demonstrated a faster and more consistent healing curve compared to the FVF group. These findings highlight the functional and aesthetic superiority of the SPBRA method when carefully evaluating both surgical techniques (Figure 2).

**Figure 3:**
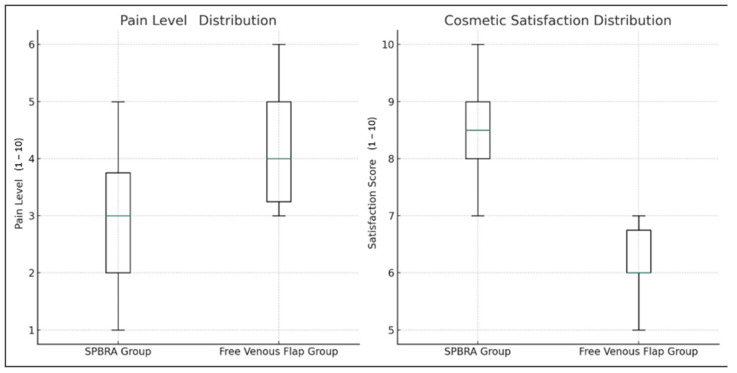
Distribution of pain levels and cosmetic satisfaction.

Our study compares two different surgical methods for treating finger tissue defects. The SPBRA group demonstrated significantly better outcomes in terms of flap survival, functional and aesthetic results, sensory recovery, and pain management compared to the FVF group (Figure 3).

**Figure 4:**
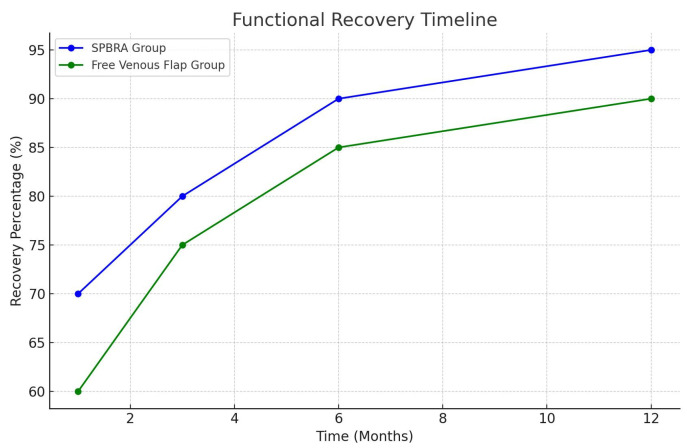
Functional recovery timeline.

Based on its consistent superiority during the healing process, the SPBRA technique can be considered the preferred option for finger repair. Its potential benefits include significant improvements in patient quality of life and long-term outcomes after surgery (Figure 4).

## 4. Discussion

Occupational accidents frequently cause finger tissue defects, leading to significant loss of manpower [7]. According to Gu et al. [8], such injuries can negatively impact individuals’ quality of life, rendering them unfit for work and limiting social integration. Finger tissue defects also substantially reduce hand function, restricting daily activities [9]. Therefore, understanding the clinical impact of these injuries is essential to develop effective treatment methods. Free flap surgery has proven to be highly effective in repairing these defects, enhancing both functional and aesthetic recovery [12]. This study compared the SPBRA and FVF techniques to evaluate their functional and aesthetic outcomes. The findings revealed that the SPBRA technique demonstrated superior flap survival rates, functional recovery, and aesthetic satisfaction compared to the FVF method [10]. These results emphasize the advantages of the SPBRA technique in improving quality of life and achieving successful long-term outcomes in patients requiring finger reconstruction [11].

In alignment with our findings, previous studies have also highlighted the high efficacy of the SPBRA technique. Fang et. al. [11]. reported that among four types of free flaps used for finger reconstruction, SPBRA provided optimal sensory recovery and aesthetic satisfaction. These findings further support the preference for SPBRA in complex finger defect repairs, showcasing its superiority in improving functionality and patient satisfaction.

The literature provides insights into the risk factors and success rates associated with free flap surgery and its complications. Common issues such as flap loss, infection, and hemorrhage are frequently observed during free tissue transfer and can directly impact surgical outcomes. The findings of this study align with these general observations, showing similar flap survival rates between the SPBRA and FVF groups, but with fewer complications reported in the SPBRA group [13,14]. Analysis of flap failure rates indicates that there may be numerous factors that influence flap survival. Specifically, reasons such as flap selection, patient comorbidities, and technical difficulties experienced during surgery can increase the risks [14]. Furthermore, venous thrombosis has been identified to contribute to flap loss, while operative strategies are important in reducing these complications [13]. These studies provide valuable insights into the challenges and potential risks associated with free flap surgery, emphasizing the need for continuous improvement in surgical procedures and management techniques. Based on this information, it can be concluded that while the SPBRA and FVF groups share similar success rates and challenges, the SPBRA technique is associated with fewer complications.

In hand surgery, functional recovery and aesthetic satisfaction are essential measures for comparing different methods. The Michigan Hand Outcomes Questionnaire is one of the tools commonly used to evaluate these aspects in patients undergoing hand surgeries. In our study, we compared the SPBRA and FVF groups in terms of functional recovery and aesthetic satisfaction. The SPBRA group demonstrated results consistent with similar findings reported in the literature [15,16]. Patient satisfaction rates were high for functional and aesthetic indications after finger pad defects and pulp repair using a palmar pivot flap [16]. The study concluded that cold intolerance and appearance improved significantly with palmar pivot flaps. In the report of Kucukguven et al. (2022), it was reported that the first dorsal metacarpal artery flap could be used for thumb defect repairs with almost the same aesthetic and functional results [15]. Based on these two studies, it can be said that flap techniques are good in terms of functional and aesthetic results.

Two-point discrimination (2PD) is recognized as an objective method for evaluating sensory recovery and measuring touch sensation. These tests are crucial for assessing sensory recovery after hand and finger surgeries. Our findings showed that the SPBRA group achieved the best performance in these tests, demonstrating its superiority over the FVF group in terms of sensory recovery. According to the literature, the choice of surgical technique, particularly the type of flap, is critical as it significantly influences the extent of sensory recovery, even when similar operations are performed under identical conditions by different surgeons. Static and moving two-point discrimination tests have been described as valid tools for assessing sensory recovery after hand amputation [17]. These tests are essential tools for managing complex hand injuries. Usami et al. discovered that certain flaps facilitate faster recovery compared to others, as demonstrated in their comparison of different flap techniques and their effects on sensory recovery [18]. Therefore, the selection of flaps remains crucial mainly during the restoration of feeling.

Various treatments were employed to repair nerves, utilizing different surgical methods for functional restoration while also evaluating aesthetic comfort and cold intolerance. The SPBRA group showed significant improvements compared to the FVF group, with better functional outcomes and higher aesthetic satisfaction among patients treated with SPBRA. Additionally, this group demonstrated superior results in tests measuring the return to normal sensory perception. However, determining whether these improvements were statistically significant requires further reference to the findings discussed by Liu et al., who highlighted the significant effects of different flap techniques on functional recovery and aesthetic outcomes [19]. They noted that both SPBRA and FVF procedures have unique benefits; however, SPBRA offers clear advantages over other methods in terms of aesthetics and functional improvements. Additionally, the researchers observed fewer complaints about cold intolerance among patients treated with the SPBRA technique compared to other methods.

The results of the surgical procedures were used to compare the two groups. The SPBRA group demonstrated a three-point advantage over the FVF group, with a flap survival rate of 94%. These findings align with similar studies in the literature. For instance, one study comparing various flap techniques reported that SPBRA resulted in lower pain scores and higher satisfaction ratings, indicating greater comfort and satisfaction among patients after treatment [20,21]. Ahmadi et al. compared free flap survival rates between end-to-end and end-to-side microvascular anastomosis and found similar survival rates in both groups. Similarly, in the investigation by Choi et al., no significant differences were observed in venous patency or flap survival across different recipient vein systems [20,21].

### Limitations of the Study

This study has several limitations. First, the relatively small sample size may limit the generalizability of the findings to a broader population. Additionally, the unequal gender distribution, with a predominance of male participants, restricts the applicability of the results to female patients. Another limitation is the retrospective design of the study, which may introduce biases compared to a prospective randomized controlled trial. Finally, the limited data on factors contributing to flap loss, such as infection or poor circulation, highlight the need for further investigation to understand these complications in greater detail. These limitations should be addressed in future studies to strengthen the findings and provide more comprehensive insights.

## 5. Conclusions

This study aimed to evaluate the functional and aesthetic outcomes of finger tissue defect repairs using the SPBRA and FVF methods. The results demonstrated that the SPBRA technique outperformed the FVF procedure, particularly in terms of flap viability, functional recovery, and aesthetic outcomes. Additionally, patients treated with the SPBRA method experienced reduced pain intensity during examinations and reported an improved quality of life compared to those who underwent FVF repair. These findings may guide the development of new surgical strategies and suggest alternative approaches for managing similar conditions in the future. Further research with larger sample sizes and long-term follow-up is recommended to validate these results and explore their broader implications.

## Figures and Tables

**Table 1:** Patient demographic information.

Features	SPBRA Group (N = 21)	FVF Group (N = 23)	Total (N = 44)
Average age	36	34	35
Gender			
Male	20	22	42
Female	1	1	2
Number of fingers treated			
1 finger	11	12	23
2 fingers	6	7	13
3+ fingers	4	4	8
Age range	18–57	18–57	18–57

**Table 2:** Surgical outcomes, flap survival rates, and complications.

Defect Characteristics	Group	Number of Patients	Average Surgical Time	Flap Survival Rate	Complications
Minimal defect (1–10 mm)	SPBRA	7	140 min	100% (0 loss)	Infection: 0, Hematoma: 0
Medium defect (11–20 mm)	SPBRA	9	165 min	88.9% (1 loss)	Infection: 1, Hematoma: 1, Flap Loss: 1
Large defect (>20 mm)	SPBRA	5	190 min	80% (1 loss)	Infection: 1, Hematoma: 0
Minimal defect (1–10 mm)	FVF	8	130 min	100% (0 loss)	Infection: 0, Hematoma: 0
Medium defect (11–20 mm)	FVF	10	150 min	90% (1 loss)	Infection: 2, Hematoma: 1
Large defect (>20 mm)	FVF	5	175 min	80% (1 loss)	Infection: 1, Hematoma: 1, Joint Contractures: 2, Keratolysis: 2

**Table 3:** Functional and aesthetic results.

Features	SPBRA Group (N = 21)	FVF Group (N = 23)
Functional Recovery Scores (Michigan Hand Outcomes Questionnaire)	85/100	80/100
1. Mobility	Good	Moderate
2. Grip Strength	Good	Moderate
3. Sensory Recovery	Good	Moderate
4. Pain Management	Moderate	Poor
Aesthetic Satisfaction Ratings	High: 15, Medium: 4, Low: 2	High: 12, Medium: 8, Low: 3
Cold Intolerance Ratings	None: 10, Mild: 6, Moderate: 3, Severe: 2	None: 5, Mild: 8, Moderate: 6, Severe: 4

**Table 4:** Sensory recovery evaluations.

Test Types	SPBRA Group (N = 21)	FVF Group (N = 23)
Static 2-Point Discrimination (S2PD)	6 mm	8 mm
Mobile 2-Point Discrimination (M2PD)	4 mm	6 mm
Semmes–Weinstein Monofilament (SWM)	2.83 g	3.61 g

**Table 5:** Statistical analysis results.

Measurement Category	Statistical Test	*p*-Value	Comparison
Functional Recovery Scores	Independent *T*-Test	0.04	SPBRA > FVF
Aesthetic satisfaction ratings	Chi-Square Test	0.03	SPBRA > FVF
Cold intolerance ratings	Mann–Whitney U Test	0.05	FVF > SPBRA
Sensory Recovery (S2PD)	Independent *T*-Test	0.01	SPBRA > FVF
Sensory Recovery (M2PD)	Independent *T*-Test	0.02	SPBRA > FVF
Sensory Recovery (SWM)	Mann–Whitney U Test	0.01	SPBRA > FVF

## Data Availability

The original contributions presented in the study are included in the article; further inquiries can be directed to the corresponding author.

## References

[1] Siotos C., Ibrahim Z., Bai J., Payne R.M., Seal S.M., Lifchez S.D., Hyder A.A. (2018). Hand injuries in low- and middle-income countries: Systematic review of existing literature and call for greater attention. Public Health.

[2] Sahu R.K., Kala P.C., Dixit P.K., Chakraborty S.S., Suresh K., Katrolia D. (2020). Finger pulp reconstruction with thenar flap: Aesthetic and functional outcome. Chin. J. Traumatol..

[3] Tu Q., Liu S., Chen T., Li S., Yan H., Li Z. (2019). A Comparative Study of Finger Pulp Reconstruction Using Free Distal Ulnar Artery Perforator Flaps and Reverse Dorsal Homodigital Island Flaps. Ann. Plast. Surg..

[4] Atilgan N., Ipek B., Duman N., Orhan O., Yilmaz M. (2023). Can anterolateral thigh flap be a rescuer in lower extremity injuries?. Eur. Rev. Med. Pharmacol. Sci..

[5] Bamba R., Malhotra G., Bueno R.A., Thayer W.P., Shack R.B. (2018). Ring Avulsion Injuries: A Systematic Review. Hand.

[6] Gurger M., Yilmaz M., Yilmaz E., Altun S. (2018). Volar percutaneous screw fixation for scaphoid nonunion. Niger. J. Clin. Pract..

[7] Jeon B.J., Yang J.W., Roh S.Y., Ki S.H., Lee D.C., Kim J.S. (2013). Microsurgical reconstruction of soft-tissue defects in digits. Injury.

[8] Gu J.X., Regmi S., Zhang N.C., Liu H.J., Zhang W.Z., Xu T. (2016). Second toe microsurgical free-flap for aesthetic and sensory reconstruction of palmar soft tissue defects of fingers. J. Plast. Reconstr. Aesthet. Surg..

[9] Iwuagwu F.C., Orkar S.K., Siddiqui A. (2015). Reconstruction of volar skin and soft tissue defects of the digits including the pulp: Experience with the free SUPBRA flap. J. Plast. Reconstr. Aesthet. Surg..

[10] Fang J., Li J., Gao J., Zhang W. (2020). Comparison of therapeutic efficacy between venous flap and the flap with superficial palmar branch of radial artery in reconstruction of the defect of finger with segmental defect of proper digital artery. Chin. J. Microsurg..

[11] Fang J., Zhang W., Song Z., Liu B., Xie C. (2019). The experience of the free superficial palmar branch of the radial artery perforator flap application. Injury.

[12] Wang L., Fu J., Li M., Han D., Yang L. (2013). Repair of hand defects by transfer of free tissue flaps from toes. Arch. Orthop. Trauma. Surg..

[13] Chiu Y.H., Chang D.H., Perng C.K. (2017). Vascular Complications and Free Flap Salvage in Head and Neck Reconstructive Surgery: Analysis of 150 Cases of Reexploration. Ann. Plast. Surg..

[14] Fischer J.P., Wink J.D., Nelson J.A., Cleveland E., Grover R., Wu L.C., Levin L.S., Kovach S.J. (2013). A retrospective review of outcomes and flap selection in free tissue transfers for complex lower extremity reconstruction. J. Reconstr. Microsurg..

[15] Kucukguven A., Uzun H., Aksu A.E. (2022). Evaluation of versatility and outcomes of the first dorsal metacarpal artery flap in thumb defects. Ulus. Travma Acil Cerrahi Derg..

[16] Ni F., Appleton S.E., Chen B., Wang B. (2012). Aesthetic and functional reconstruction of fingertip and pulp defects with pivot flaps. J. Hand Surg. Am..

[17] Chua M., Seth I., Rozen W.M. (2024). The reliability and applicability of the Ten Test in hand injuries: A systematic review. Hand Ther..

[18] Usami S., Kawahara S., Yamaguchi Y., Hirase T. (2015). Homodigital artery flap reconstruction for fingertip amputation: A comparative study of the oblique triangular neurovascular advancement flap and the reverse digital artery island flap. J. Hand Surg. Eur. Vol..

[19] Liu Y., Jiao H., Ji X., Liu C., Zhong X., Zhang H., Ding X., Cao X. (2014). A comparative study of four types of free flaps from the ipsilateral extremity for finger reconstruction. PLoS ONE.

[20] Ahmadi I., Herle P., Miller G., Hunter-Smith D.J., Leong J., Rozen W.M. (2017). End-to-End versus End-to-Side Microvascular Anastomosis: A Meta-analysis of Free Flap Outcomes. J. Reconstr. Microsurg..

[21] Choi J.W., Kim Y.C., Jeon D.N., Jeong W.S., Koh K.S., Oh T.S., Eom J.S., Kim E.K., Hong J.P., Suh H.P. (2020). Impact of Recipient Vein Selection on Venous Patency and Free Flap Survival in 652 Head and Neck Reconstructions. J. Reconstr. Microsurg..

